# Effect of Resveratrol on Reactive Oxygen Species-Induced Cognitive Impairment in Rats with Angiotensin II-Ind   uced Early Alzheimer’s Disease †

**DOI:** 10.3390/jcm7100329

**Published:** 2018-10-05

**Authors:** Yu-Te Lin, Yi-Chung Wu, Gwo-Ching Sun, Chiu-Yi Ho, Tzyy-Yue Wong, Ching-Huang Lin, Hsin-Hung Chen, Tung-Chen Yeh, Chia-Jung Li, Ching-Jiunn Tseng, Pei-Wen Cheng

**Affiliations:** 1 Section of Neurology, Kaohsiung Veterans General Hospital, Kaohsiung 81300, Taiwan; ytlin@vghks.gov.tw (Y.-T.L.); Chlin2524@vghks.gov.tw (C.-H.L.); 2 Center for Geriatrics and Gerontology, Kaohsiung Veterans General Hospital, Kaohsiung 81300, Taiwan; 3 Section of Neurology, Zouying Branch of Kaohsiung Armed Forces General Hospital Kaohsiung, Kaohsiung 81300, Taiwan; gasric61@yahoo.com.tw; 4 Department of Anesthesiology, Kaohsiung Medical University Hospital, Kaohsiung Medical University, Kaohsiung 81700, Taiwan; gcsun39@yahoo.com.tw; 5 Department of Biomedical Science, National Sun Yat-Sen University, Kaohsiung 80400, Taiwan; hochiuyi1987@gmail.com; 6 Research Assistant Center, Show Chwan Memorial Hospital, Changhua 50000, Taiwan; wongtzyyyue@gmail.com (T.-Y.W.); nigel6761@gmail.com (C.-J.L.); 7 Department of Medical Education and Research, Kaohsiung Veterans General Hospital, Kaohsiung 81300, Taiwan; Shchen0910@gmail.com; 8 Department of Internal Medicine, Division of Cardiology, Kaohsiung Veterans General Hospital, Kaohsiung 81300, Taiwan; tcyeh9@gmail.com; 9 Department of Pharmacology, Medical Research, China Medical University Hospital, China Medical University, Taichung 40400, Taiwan; 10 Yuh-Ing Junior College of Health Care & Management, Kaohsiung 82100, Taiwan; 11 Shu-Zen Junior College of Medicine and Management, Kaohsiung 80700, Taiwan

**Keywords:** Alzheimer’s disease, central nervous system, hypertension, brain-derived neurotrophy factor, NADPH oxidase

## Abstract

Recent studies have indicated that several anti-hypertensive drugs may delay the development and progression of Alzheimer’s disease (AD). However, the relationships among AD, hypertension, and oxidative stress remain to be elucidated. Here, we aimed to determine whether reactive oxygen species (ROS) reduction by resveratrol in the brain leads to cognitive impairment reduction in rats with angiotensin II (Ang-II)-induced early AD. Male Wistar Kyoto (WKY) rats with Ang-II-induced AD were treated with losartan or resveratrol for two weeks. Our results show decreased blood pressure, increased hippocampal brain-derived neurotrophic factor (BDNF) level, and decreased nucleus tractus solitarius (NTS) ROS production in the Ang-II groups with losartan (10 mg/kg), or resveratrol (10 mg/kg/day) treatment. Furthermore, losartan inhibition of hippocampal Tau^T231^ phosphorylation activated Akt^S473^ phosphorylation, and significantly abolished Ang-II-induced Aβ precursors, active caspase 3, and glycogen synthase kinase 3β (GSK-3β)^Y216^ expressions. Consistently, resveratrol showed similar effects compared to losartan. Both losartan and resveratrol restored hippocampal-dependent contextual memory by NADPH oxidase 2 (NOX2) deletion and superoxide dismutase 2 (SOD2) elevation. Our results suggest that both losartan and resveratrol exert neuroprotective effects against memory impairment and hippocampal damage by oxidative stress reduction in early stage AD rat model. These novel findings indicate that resveratrol may represent a pharmacological option similar to losartan for patients with hypertension at risk of AD during old age.

## 1. Introduction

Alzheimer’s disease (AD) is an age-dependent neurodegenerative disorder associated with abnormal energy metabolism, representing the most common neurodegenerative disorder worldwide [1,2]. Alzheimer’s disease is characterised neuropathologically by the formation of senile plaques and neurofibrillary tangles, and clinically by the progressive deterioration of memory and other cognitive functions [3] which cannot be prevented using any available treatments [4]. The pathogenesis of AD is a multifactorial neurodegenerative process with several damaged pathways due to oxidative stress injury, abnormal energy processing, mitochondrial dysfunction, and inflammation [5,6]. Moreover, evidence has shown that hypertension is a major risk factor for the AD development during aging [7]. The nucleus tractus solitarius (NTS) in the brainstem dorsal medulla is the primary integrating centre for cardiovascular regulation, as well as other central nervous systems (CNS) autonomic functions. Furthermore, increase in circulating and brain levels of the primary effector peptide of the renin–angiotensin–aldosterone system, angiotensin II (Ang-II), plays important roles in arterial hypertension, as well as the AD pathophysiology [8]. Therefore, a trio relationship exists among hypertension, cerebrovascular disease, and decreased cognitive function—an index of hippocampal dysfunction [9]. Numerous pre-clinical and clinical renin–angiotensin system (RAS) in AD research may be able to explain the connection between hypertension and AD [10]. Kehoe [10] explained that, although Angiotensin-converting enzyme (ACE) is normally abundant in neuronal density, ACE activity was higher in typical AD patients despite significant neuronal loss. Furthermore, they found that the elevated ACE activity correlated positively with the severity of tau pathology [11].

Ang-II binds excessively to the AT1 receptor which negatively affects cognition by inhibiting hippocampal long-term potentiation (LTP) and enhancing β-amyloid production [12,13]. The abnormal binding may lead to widespread neuronal injury such as increased reactive oxygen species (ROS), activated NADPH oxidases, compromised blood–brain barrier (BBB), altered nutrient uptake, and β-amyloid toxicity in the cerebral vasculature [12,14,15].

Resveratrol (3,5,4′-trihydroxy-trans-stilbene) is a stilbenoid, a type of natural phenol that may have beneficial effects for human diseases such as type 2 diabetes, obesity, and cancer [16]. Resveratrol facilitates anti-amyloidogenic cleavage of β-amyloid precursor protein, promotes clearance of neurotoxic Aβ peptides, and reduces oxidative stress to protect neurons [17,18]. Accumulating evidence indicates that resveratrol attenuates oxidative imbalance by simultaneously enhancing ROS production and downregulating key antioxidant enzymes such as copper-zinc superoxide dismutase (SOD1) and manganese superoxide dismutase (SOD2) [19]. Previously, we demonstrated that resveratrol abolishes ROS generation and enhances SOD2 expression, negatively regulating Racl-induced NADPH oxidase levels in the NTS during oxidative stress-associated hypertension [20]. Previous studies further suggest that oxidative imbalance and neuronal damage play a critical role in AD initiation and progression [21]. In addition, NAD(P)H oxidase plays a role in multiple central autonomic networks associated with Ang-II-induced ROS in neurons [22]. We previously observed significant decreased systolic blood pressure (SBP) and ROS generation in the NTS of hypertensive rats (SHRs) treated with losartan or tempol for two weeks [23]. Here, we aimed to examine whether resveratrol treatment reduces brain ROS to ameliorate cognitive impairment in rats with Ang-II-induced early AD. Results revealed that two-week resveratrol treatment decreased blood pressure (BP), increased hippocampal brain-derived neurotrophic factor (BDNF) level, and decreased NTS ROS production in the Ang-II groups. Taken together, our result indicates that resveratrol exerts neuroprotective effects similar to losartan in memory impairment and hippocampal damage in a rat model of AD early stage by oxidative stress reduction.

## 2. Materials and Methods

### 2.1. Experimental Chemicals

All experimental chemicals were purchased from Sigma-Aldrich (St. Louis, MO, USA), unless otherwise indicated.

### 2.2. Animals

Ten-week-old male Wistar Kyoto (WKY) rats were obtained from the National Science Council Animal Facility (NSCAF; Taipei, Taiwan) and housed in the animal room of Kaohsiung Veterans General Hospital (VGHKS; Kaohsiung, Taiwan). Both NSCAF and VGHKS are internationally certified by the Association for Assessment and Accreditation of Laboratory Animal Care (AAALACi). Rats housed in the animal room of VGHKS were fed in a specific pathogen-free (SPF) room. SPF facilities are designed to maintain rodents in an environment that is free of certain infectious organisms that are pathogenic and/or capable of interfering with research objectives. The rats were kept in individual cages in a light-controlled room (12-h light/12-h dark cycle), the temperature of which was maintained between 23 °C and 24 °C. The rats were given normal rat chow (Purina; St. Louis, MO, USA) and tap water ad libitum. All animal research protocols were approved by the Animal Research Committee, and the institutional review board at VGHKS approved all study procedures (VGHKS-2018-A002). The study was performed in accordance with approved guidelines and conducted in compliance with the Declaration of Helsinki.

The rats were housed in an animal room at VGHKS, randomly divided into four groups with six rats in each group: (i) the sham group received an intracerebroventricular (ICV) injection of artificial cerebrospinal fluid (aCSF) and an oral dose (1 mL/kg body weight (BW)) of distilled water once every 24 h (vehicle control); (ii) the WKY + Ang-II group received an ICV injection of angiotensin II (14.4 μg/μL) and an oral dose of distilled water; (iii) the WKY + Ang-II + losartan group was treated with losartan (10 mg/kg BW, Chunghwa Yuming Healthcare, Kaohsiung, Taiwan) and received an ICV injection of angiotensin II; and (iv) the WKY + Ang-II + resveratrol group was treated with resveratrol (10 mg/kg BW) and received an ICV injection of angiotensin II. All surgeries were performed in an effort to minimise animal suffering.

After 14 days, rats were euthanised using CO_2_ in accordance with the 2013 AVMA Guidelines for the Euthanasia of Animals. All rats were killed using 100% CO_2_, with death occurring within 2–5 min. Brains were immediately dissected on ice, frozen, and maintained at −20 °C until assayed.

### 2.3. Intracerebroventricular Injection Procedure

Intracerebroventricular infusion experiments were performed following a stabilisation period of at least 30 min after microinjector insertion into the ventricular-guided cannula. Blood pressure was monitored for three days after drug infusion. As vehicle control, we analysed the effect of an ICV injection of aCSF (142 mmol/L NaCl, 5 mmol/L KCl, 10 mmol/L glucose, and 10 mmol/L HEPES, pH 7.4). Ang-II (14.4 μg/μL/per day). Resveratrol was initially dissolved in DMSO and then diluted in ddH_2_O at a final concentration of 1% DMSO. Basal blood pressure was examined prior to injection. The daily ICV drug infusions were performed over a 2 min period and delivered as a single bolus with final volume of 5 μL from Day 0 to Day 14.

### 2.4. Blood Pressure Measurement

Using a previously described tail-cuff method (CODA, Kent Scientific Corporation, Torrington, CT, USA), we measured SBP and heart rate prior to losartan or resveratrol treatment (Day 0). Measurements were also obtained 3, 7, 11, and 14 days after surgery, as well as prior to sacrifice. All measurements were performed between 08:00 and 24:00. In this method, the pulsation reappearance on the digital display of the BP cuff detected by pressure transducer was amplified and recorded as the SBP. During measurement, we obtained a rapid series of ten individual readings, the highest and lowest were discarded, and the remaining eight were averaged.

### 2.5. Morris Water Maze 

Cognitive and behavioural functions were assessed using the Morris Water Maze (MWM) task. Briefly, a circular pool (diameter: 180 cm) was filled with tap water maintained at 24 ± 1 °C, with an escape platform (diameter: 12 cm) submerged 2 cm below water surface. Each rat underwent four trials per day over four consecutive days (Day 3 to Day 6). For each trial, the rat was placed in the pool (facing pool wall) at one of four selected starting points (north, south, east, or west pole). Upon locating the platform, the rat was allowed to remain there for 20 s before being returned to its cage. If the rat did not find the platform within 90 s, the time was recorded as 90 s, following which the rat was guided to the platform and allowed to remain there for 20 s. The escape latency and swim speed were measured using a video tracking system (EthoVision XT, Noldus, Leesburg, VA, USA). After the last training trial, rats were subjected to a probe trial in which the platform was removed. Animals were placed in the pool at the same pole and allowed to swim for 2 min. The time that each animal spent in the quadrant that had previously contained the hidden platform was recorded.

### 2.6. Measurement of Aβ40 and Aβ42 in the Hippocampus and Post-Hypothalamus

We measured soluble Aβ40 and Aβ42 levels in the hippocampus and post-hypothalamus using Ultrasensitive Rat Aβ40 and Aβ42 ELISA kits (Arigo Biolaboratories Corp, Hsinchu, Taiwan), in accordance with manufacturer instructions. Expression levels were detected using a Biochrom Anthos Zenyth 200rt Microplate Reader (Cambridge, UK).

### 2.7. Immunoblotting Analysis

Total protein was prepared by homogenising hippocampal and post-hypothalamic tissues in lysis buffer containing a protease inhibitor cocktail and a phosphatase inhibitor cocktail. The sample was subsequently incubated for 1 h at 4 °C. The protein extracts (20 μg/sample based on the Bicinchoninic Acid (BCA) protein assay, Pierce Chemical Co., Rockford, IL, USA) were resolved on a 6% polyacrylamide gel and transferred to a PVDF membrane (GE Healthcare, Buckinghamshire, UK). The membranes were incubated in the appropriate anti-P-Tau^T231^ (ab151559), anti-P-Akt^S473^ (4060, Cell Signaling Technology, Beverly, MA, USA), anti-P-GSK-3bS9 (05-643, EMD Millipore, Billerica, MA, USA), anti-P-GSK-3β^Y216^ (ab75745) antibodies at 1:1000 in PBST with 5% BSA at 4 °C overnight, anti-amyloid precursor protein (ab12266), anti-gp91-phox (sc5827, Santa Cruz Biotechnology, Dallas, TX, USA), anti-Tau (ab80579), anti-Akt (9272), anti-p67-phox (sc7663), anti-GSK-3β (07-389), anti-p47-phox (sc14015), anti-p22-phox (sc11712), or anti-caspase 3 (9662) antibodies at 1:1000 in PBST with 5% BSA at room temperature (RT) for 1 h. The membranes were then incubated in an HRP-labelled goat anti-rabbit secondary antibody at 1:10,000. The membranes were developed using an ECL-Plus detection kit (GE Healthcare).

### 2.8. Immunohistochemistry Analysis

For immunohistochemistry experiments, initial specimen processing and staining were performed as described earlier [23]. Sections were first stained with avidin-biotin peroxidase (goat anti-rabbit biotin), followed by double-indirect staining with horse anti-mouse AP. Briefly, sections were incubated with primary antibodies Anti–P-TauT231 (AT-180, Thermo, Thermo Fisher Scientific, Waltham, MA, USA), anti-P-TauT181 (AT-270, Thermo), or anti-BDNF(ab226843) at 1:50 overnight at 4 °C. Slides were then washed three times with PBS, following which they were incubated for 1 h at RT with a combination of biotinylated HRP-conjugated goat anti-rabbit (1:200) secondary antibodies. After three washes with PBS, the sections were incubated with ABC complex (1:50) for 30 min at RT. After three PBS washes, sections were visualised using the DAB substrate kit (Vector Laboratories, Burlingame, CA, USA), counterstained with haematoxylin. After PBS rinse, the sections were dehydrated using a graded series of ethanol and xylene. The sections were then photographed using an Olympus microscope equipped with a Nikon Cool Scan 995 digital camera (Nikon, Tokyo, Japan).

### 2.9. Statistical Analyses

All data are expressed as mean ± standard error of the mean (SEM). Paired *t*-tests were used to compare baseline and post-treatment BP measurements, while a one-way analysis of variance (ANOVA) with Scheffe’s post hoc testing was employed to evaluated differences among the groups. The level of statistical significance was set at *p* < 0.05.

## 3. Results

### 3.1. Brain-Derived Neurotrophic Factor Levels and Superoxide Imbalance Contributed to Hypertension and Early AD

**Figure 1:**
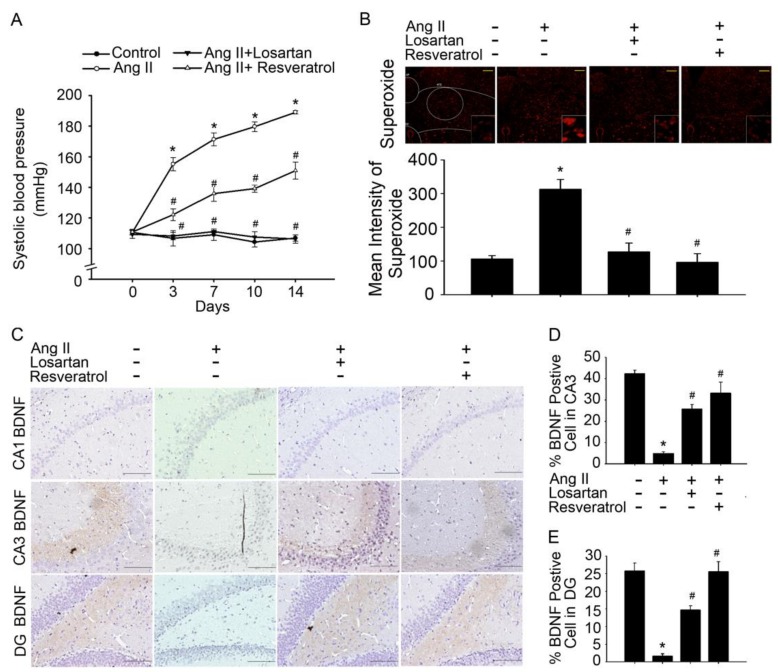
Downregulation of BDNF levels was associated with increased superoxide expression in rats with Ang-II-induced early Alzheimer’s disease (AD). (**A**) Time course of systolic blood pressure (SBP) after intracerebroventricular administration of angiotensin II (Ang-II) for two weeks. The filled circles (●) represent the Wistar Kyoto (WKY) group, the open circles (○) represent the Ang-II group, the inverted filled triangles (▼) represent the Ang-II + losartan group, and the open triangles (∆) represent the Ang-II + resveratrol group. SBP was measured on Days 0, 4, 7, 11, and 14. The data are presented as the mean ± standard error of the mean (SEM); *n* = 6. * *p* < 0.05 vs. the WKY and ^#^ *p* < 0.05 vs. the Ang-II group. (**B**) Confocal microscopy analysis of DHE-treated brain sections in the nucleus tractus solitarius (NTS) after treatment with losartan or resveratrol. Bar graph showing the superoxide production ratio after treatment with Ang-II and/or losartan or resveratrol. Note the significant decrease in Ang-II-induced superoxide production after the administration of losartan or resveratrol. (C) In situ qualitative analysis of BDNF-immunopositive cells in the hippocampus of AD model rats. Scale bar, 200 mm. (**D**,**E**) Bar graph showing BDNF-expressing cells after treatment with Ang-II and/or losartan or resveratrol. Note the significant increase in Ang-II-induced BDNF production after the administration of losartan or resveratrol. The percentage of BDNF-positive cells was determined by counting the BDNF-expressing cells in each hemisphere of the hippocampus (CA1 and CA3) at ×200 magnification. These values were divided by the total number of cells in the same paraffin section. BDNF, brain-derived neurotrophic factor; DG, dentate gyrus.

**Figure 2:**
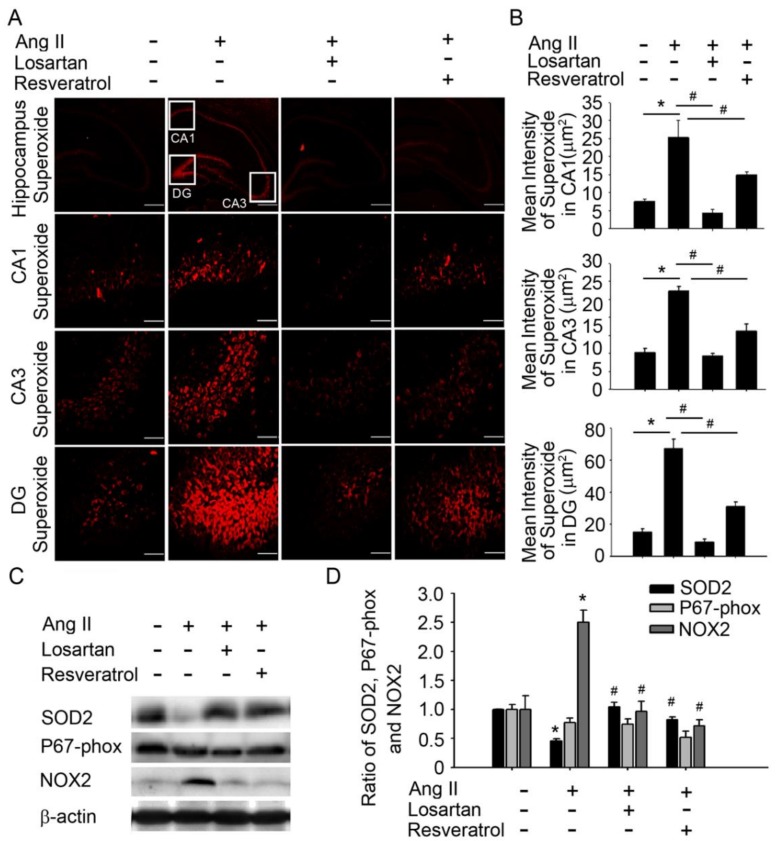
Resveratrol abolished superoxide and NOX2, and reduced SOD2 activity in the hippocampus of AD rats. (**A**) Confocal microscopy analysis of DHE-treated brain sections in the hippocampus after treatment with losartan or resveratrol. (**B**) Bar graph showing the superoxide production ratio after treatment with Ang-II and/or losartan or resveratrol. Note the significant decrease in Ang-II-induced superoxide production after the administration of losartan or resveratrol. (**C**,**D**) Quantitative immunoblot analysis demonstrating decreased expression of the NOX2 ratio in the hippocampus of Ang-II-treated rats after losartan or resveratrol treatment. The SOD2 protein level in the hippocampus was significantly increased following losartan or resveratrol treatment. The values are presented as the mean ± SEM; *n* = 6. * *p* < 0.05 vs. the WKY group. ^#^ *p* < 0.05 vs. the Ang-II group. NOX2, NADPH oxidase 2; SOD2, manganese superoxide dismutase.

Previous studies have suggested that BDNF-deficient mice are more susceptible to stress-induced oxidative damage, as indicated by the direct association between oxidative stress indicators and BDNF levels in the brain [24]. Oxidative stress is an important pathogenic factor in hypertension development. Angiotensin receptors (AT1R) play a pivotal role in the development and maintenance of hypertension during oxidative stress [25]. Thus, we aimed to determine ROS level in the NTS of Ang-II-induced AD mice, and the BDNF release in the hippocampus. In addition to increased SBP, Ang-II-induced AD rats exhibited significantly higher superoxide level in the NTS and hippocampal areas CA1, CA3, and dentate gyrus (DG) compared to those control group. Furthermore, losartan or resveratrol treatment reversed these effects (Figure 1A,B and Figure 2A,B ). Interestingly, losartan or resveratrol treatment markedly increased BDNF levels in the hippocampus and NTS of Ang-II-induced AD rats (Figure 1C and Figure S1).

To investigate whether resveratrol limits ROS production via inhibition of NADPH oxidases during Ang-II-induced early AD, we examined the expression of NADPH oxidase and SOD when both Ang-II and resveratrol were administered. Our results indicate that losartan or resveratrol abolished NADPH oxidase 2 (NOX2) expression and reduced SOD2 level in the hippocampus (Figure 2C,D). These results indicate that ROS elimination may be required to elevate BDNF levels and activate the depressor response. Taken together, these findings suggest that resveratrol mitigates oxidative stress, normalises BDNF levels, and reduces BP in Ang-II-induced early AD.

### 3.2. Resveratrol Impaired the Activity of Aβ Precursors, Active Caspase 3, and GSK-3-Tau by Normalising Renal AT1R Signalling in the Hippocampus of Rats with Ang-II-Induced Early AD

**Figure 3:**
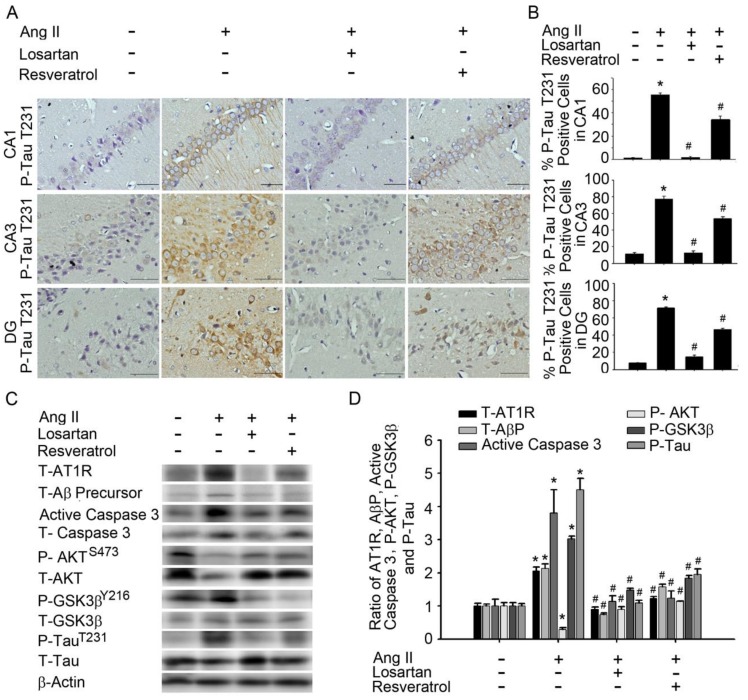
Resveratrol attenuated Ang-II-induced Aβ precursor and caspase 3-Akt-GSK-3βTau pathways in the hippocampus of AD rats. (A) In situ qualitative analysis of P-Tau^T231^-immunopositive cells in the hippocampus of AD model rats. Scale bar, 200 mm. (**B**) Bar graph showing P-Tau^T231^-expressing cells after treatment with Ang-II and/or losartan or resveratrol. Note the significant decrease in Ang-II-induced P-Tau^T231^ production after the administration of losartan or resveratrol. The percentage of P-Tau^T231^-positive cells was determined by counting the P-Tau^T231^-expressing cells in each hemisphere of the hippocampus (CA1, CA3, and DG) at ×200 magnification. These values were divided by the total number of cells in the same paraffin section. (**C**) Immunoblot demonstrating decreased levels of the proteins T-AT1R, T-Aβ precursor, T-active-caspase 3, P-GSK-3β^Y216^, and P-Tau^T231^ in the hippocampus after treatment with Ang-II and/or losartan or resveratrol. (**D**) Quantitative immunoblot analysis demonstrating reductions in T-AT1R, T-Aβ precursor, T-active-caspase 3, P-GSK-3β^Y216^, and P-Tau^T231^ expression in the hippocampus of rats with Ang-II-induced AD following treatment with losartan or resveratrol. The values are presented as the mean ± SEM; *n* = 6. * *p* < 0.05 vs. the WKY group. ^#^ *p* < 0.05 vs. the Ang-II group.

Accumulating evidence has indicated that patients with AD exhibit significantly higher levels of anti-AT1R and tau compared to healthy controls [26]. However, the angiotensin system increases BBB permeability, induces oxidative stress in the brain microcirculation, leading to Aβ increase and tau pathologies [27]. Therefore, we investigated whether resveratrol affects AT1R signalling in the hippocampus of rats with Ang-II-induced early AD. Immunohistochemistry (IHC) experiments demonstrated that losartan or resveratrol treatment influenced the expression of phosphorylated Tau^T231^ in hippocampal areas CA1, CA3, and DG in rats with Ang-II-induced AD (Figure 3A,B). Immunoblot analyses of proteins extracted from the hippocampus demonstrated that losartan or resveratrol treatment decreased the expression of AT1R, Aβ precursors, and active caspase 3 in Ang-II-induced groups. Similarly, GSK-3β^Y216^ expression and Tau^T231^ phosphorylation in the hippocampus were significantly attenuated by losartan or resveratrol treatment. The addition of losartan or resveratrol increased Akt activation, as well as hyperphosphorylation of critical Akt substrates, in Ang-II-induced groups (Figure 3C,D). Resveratrol treatment resulted in Ang-II-induced AT1R expression, Aβ precursors, active caspase 3, GSK-3, and tau activation in the hippocampus. These results suggest that resveratrol attenuated Ang-II-induced tau pathologies, improved Akt activation, and attenuated down-regulation of the AT1R–Aβ–caspase 3–GSK-3β signalling pathway.

### 3.3. Resveratrol Treatment Improved Spatial Learning and Memory in Rats with Ang-II-Induced Early AD

**Figure 4:**
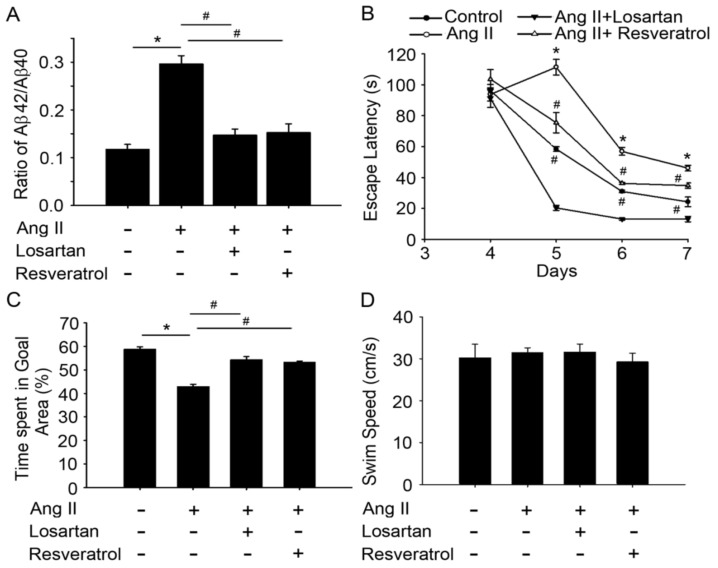
Resveratrol reversed impairments in hippocampal-dependent and contextual memory in rats with Ang-II-induced early AD. (**A**) Bar graph showing the Aβ42 production ratio after treatment with Ang-II and/or losartan or resveratrol. Note the significant decrease in Ang-II-induced Aβ42 production after the administration of losartan or resveratrol. (**B**) Bar graph showing the latency to find the hidden platform. (**C**) Bar graph showing the time spent in the central area. (**D**) Bar graph showing the swim speed. Note that learning and memory deficits were also reversed in Ang-II-treated rats after losartan or resveratrol administration. The values are presented as the mean ± SEM; *n* = 6. * *p* < 0.05 vs. the WKY rats and ^#^ *p* < 0.05 vs. the Ang-II-treated rats.

Fibrillar Aβ is the major constituent of senile plaques in the brains of AD patients. Some patients with early-onset AD showed elevated levels of Aβ42/Aβ40 [28]. To investigate the role of resveratrol in spatial learning and memory improvements during Ang-II-induced early AD, we examined the expression of Aβ42/Aβ40 and MWM results in rats treated with losartan or resveratrol. The Ang-II-induced AD rats exhibited significantly higher Aβ42/Aβ40 level in the hippocampus compared to the controls (Figure 4A, Histograms 1 and 2). Interestingly, losartan or resveratrol treatment markedly inhibited Aβ42/Aβ40 expression in the hippocampus of the rats (Figure 4A, Histograms 3 and 4). While all rats exhibited progressive decrease in escape latency during the MWM task (Figure 4B), Ang-II-treated rats took significantly longer time to reach the platform compared to the controls (*p* < 0.01). Furthermore, this increase in escape latency was significantly ameliorated by losartan and resveratrol treatment (Days 5–7, *p* < 0.05 vs. Ang-II groups). The AD rats spent significantly less time in the central area compared to the control group, whereas more time spent in this area after losartan treatment. No significant differences were observed between losartan or resveratrol treatment in the Ang-II groups (Figure 4C). The rats exhibited similar motor capabilities with no difference in swimming speed (Figure 4D), indicating that latency difference was not due to swimming speed. These results suggest that resveratrol or losartan attenuated Ang-II-induced impairments in hippocampal-dependent and contextual memory.

## 4. Discussion

According to Alzheimer’s Disease International (ADI), the number of AD patients will quadruple in India, China, other countries in Asia, Australasia, and Oceania, from approximately 16 million in 2010 to 61 million by 2050. The total worldwide costs of dementia in 2010 were estimated to be $604 billion (USD) (of which 70% occur in Europe and North America), approximately 1% of global gross domestic product [29].

**Figure 5:**
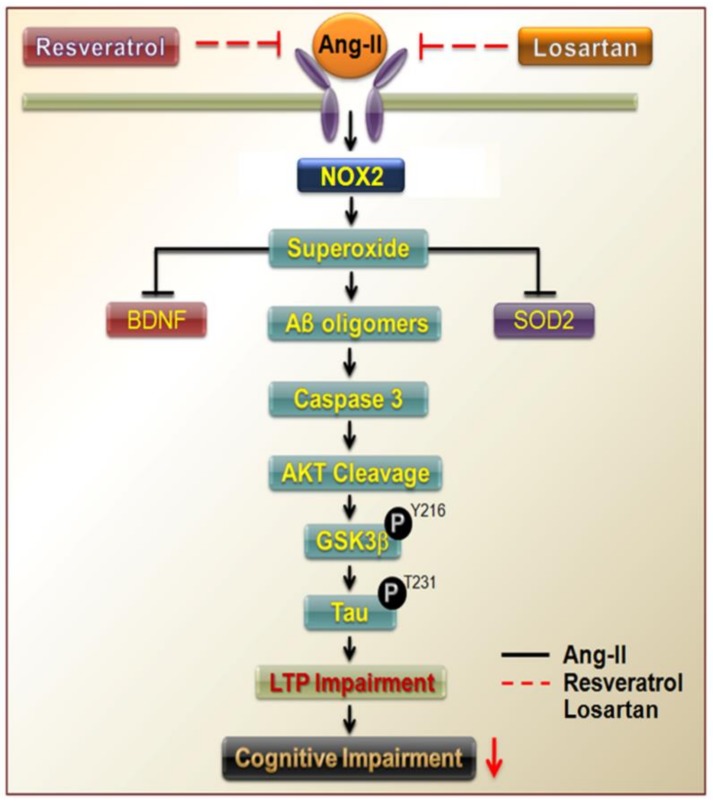
Resveratrol attenuated ROS-induced cognitive impairments in rats with Ang-II-induced early AD. Ang-II not only increased the generation of superoxide and the activity of the Aβ–caspase 3–Akt–GSK-3β–Tau pathway by positively regulating NOX2 levels, but also attenuated SOD2 and BDNF expression in the hippocampus (black line). However, treatment with losartan or resveratrol improved cognitive impairments in rats with Ang-II-induced early AD by abolishing ROS generation and reducing activity of the Aβ–caspase 3–Akt–GSK-3β–Tau pathway by negatively regulating NOX2 levels (red line). ROS, reactive oxygen species.

A previous population-based study has revealed that hippocampal atrophy (HA) is usually attributed to the neurofibrillary tangles and neuritic plaques associated with AD [30]. Several studies have indicated that the clinical end points of AD are strongest in those who have never been treated for hypertension. Additional studies have demonstrated that treatment with antihypertensive medication reduces the risk associated with high BP [31,32]. Here, we observed that central BP is regulated by ROS levels in the NTS, which are thought to contribute to down-regulation of BDNF expression in both the NTS and hippocampus of rats with Ang-II-induced AD. In addition, our results suggest that resveratrol not only attenuated increases in superoxide levels in the NTS and increased BDNF expression, but also increased the antioxidant capacity of the NTS in rats with hypertension. Our findings further demonstrated that Ang-II increased the generation of superoxide and the activity of the Aβ–caspase 3–Akt–GSK-3β-Tau pathway by positively regulating NOX2 levels. Such changes were also accompanied by decreases in SOD2 and BDNF expression in the hippocampus. However, treatment with losartan or resveratrol improved cognitive function in rats with Ang-II-induced early AD by abolishing ROS generation and reducing activity of the Aβ–caspase 3–Akt–GSK-3β-Tau pathway activity by negatively NOX2 levels. Therefore, our findings suggest that early treatment with resveratrol lowers oxidative stress, preserves SOD function, and attenuates hypertension development (Figure 5).

Studies involving patients with hypertension have revealed that high BP is associated with atrophy of the hippocampus and temporal lobe, as well as an increased risk of cognitive decline, suggesting that such patients are at increased risk of developing AD [30]. Indeed, neurofibrillary tangles, senile plaques, and neuronal lesions have been observed in patients with hypertension [33]. While such findings suggest a relationship among hypertension, cerebrovascular disease, and decreased cognitive function [9], it remains to be determined whether hippocampal changes are the consequences of pre-existing hypertension, or whether hypertension and brain pathology reflect a central defect in patients with AD [34].

Some studies have suggested that SHRs and rats with Deoxycorticosterone acetate (DOCA)-salt-induced hypertension exhibit low BDNF expression and deficient neurogenesis in the hippocampus. However, treatment with oestrogens may normalise brain parameters (i.e., BDNF levels) by decreasing peripheral BP in both rat groups [35,36].

Losartan or resveratrol normalised BP and superoxide levels in the NTS, increased BDNF levels in the hippocampus and NTS of Ang-II-induced AD rats (Figure 1D,E and Figure S1). These results indicate that ROS elimination may be required to increase BDNF levels and activate the depressor response. Our study provides convincing evidence that resveratrol attenuates AT1R-induced ROS generation, decreases Aβ and tau pathologies, and improves cognitive function in Ang-II-induced early AD rats. ROS in brain has been attributed to neuropathogenesis of hypertension via enhancement of sympathetic nervous system activity. Oxidative stress can be defined as increased bioactivity of ROS relative to antioxidant defences [37], and nitric oxide (NO) bioavailability is key to oxidative stress [38,39]. Consistent with our previous findings that resveratrol decreases BP better than rosuvastatin, abolishes ROS generation, and enhances activity of the ERK1/2-RSK-nNOS pathway by activating 5′ AMP-activated protein kinase (AMPK) to negatively regulate Racl-induced NADPH oxidase levels in the NTS during oxidative stress-associated hypertension [40]. Besides, resveratrol suppressed AT1R expression through sirtuin 1 (SIRT1) activation both in vivo and in vitro [41]. Resveratrol suppression of angiotensin II-induced hypertension is in part due to inhibited renin–angiotensin system [42]. In contrast to ACE, ACE2 appears to modulate hypertension development through regulation of angiotensin II type 2 receptor (AT2R) and Mas receptor expressions. It is well known that over activation of the classical RAS leads to hypertension [43]. Conversely, early inhibition of the classical RAS has been shown to prevent the development of hypertension [44]. Recent evidence suggests that resveratrol can ameliorate most of the features of MetS and the beneficial effects of resveratrol treatment are commonly associated with down-regulation of the classical RAS axis and stimulation of the alternative RAS axis [41].

More recently, several potential mechanisms of resveratrol’s neuroprotective effects have been found. Several studies in cell and animal models have demonstrated that resveratrol exhibits anti-inflammatory and antioxidant effects, inhibits beta-amyloid (Aβ) protein aggregation and modulates intracellular effectors involved in neuronal cell survival/death [45]. Vingtdeux et al. showed that resveratrol activated AMPK by increasing intracellular calcium levels and that the inhibition of AMPK counteracted the effect of resveratrol on Aβ levels [46]. This effect was also obtained by in vivo, wherein peripheral administration of resveratrol activated AMPK and reduced cerebral Aβ levels and accumulation in the mouse cerebral cortex, likely via SIRT-independent pathway. It has been reported that activation of sirtuins, particularly SIRT-1, may protect neurons against apoptosis, inflammation and oxidative stress, suggesting that these enzymes might be new targets for treating neurological disorders such as stroke, AD and Parkinson's disease (PD) [47]. Nicotinamide, a SIRT1 inhibitor, attenuated the protective action of resveratrol against Aβ^25–35^-induced toxicity. Resveratrol has been reported to bind directly to Aβ1–42, interferes with in Aβ^1–42^ aggregation, changes Aβ^1–42^ oligomer conformation and attenuates Aβ^1–42^ oligomeric cytotoxicity [48]. Another group suggested that it remodels three Aβ conformers (i.e., soluble oligomers, fibrillar intermediates, and amyloid fibrils) into aggregated forms that are non-toxic [49]. Sinha et al. reported that in vivo protective action of resveratrol (5–300 mg/kg) was observed in transgenic AD models [50].

Currently, convergence of various evidence has now positioned the RAS as a potential target for AD intervention [51]. With increased availability of RAS acting drugs, clinical trials have now begun to explore various the role of RAS in AD development and pathology; for example, Phase II multi-centre RADAR trial of losartan in hypertensive and normotensive AD patients (Study ISRCTN93682 878 at http://www.isrctn.com) with outcome MRI-based of brain structure and volume after 12 months of treatment [10]. Besides, a clinical study is under way for resveratrol evaluation as a dietary ingredient in mild to moderate AD. A Phase I, randomized placebo controlled pilot study is under way to determine safety of resveratrol (250–1000 mg/day; for 12–52 weeks) supplementation, memory and physical performance in older adults (ClinicalTrials.gov Identifier: NCT01126229). However, analogue development is very challenging due to poor absorption of resveratrol. Recently, resveratrol analogues such as pterostilbene has been synthesized and is effective in treating neurotoxicity of rodent models [52]. Moreover, nanotechnology advancement has been shown to improve polyphenol solubility and stability in rodents. The encapsulation of resveratrol by lipid-core nanocapsules improves resveratrol efficacy. These new technologies ought to be used to confirm if resveratrol is a potential therapeutic agent in age-related neurodegenerative disorders. In fact, losartan is now generically available, resveratrol-based drug development would be challenged by production cost, as well as marketing. However, recent clinical trials showed that resveratrol supplementation affected neuroinflammation and Aβ deposition with high variations of plasma concentration. The reduced Aβ40 is determined in both CSF and plasma to a lesser extent in the resveratrol treatment group compared to placebo group, suggesting a central effect through blood–brain barrier penetration [53]. Notably, resveratrol is health beneficial, may serve as an alternative when consumed as whole, or as supplements rich in phytochemicals such as resveratrol.

Treatment of vascular risk factors has been associated with a reduced AD occurrence and slower cognitive decline in AD patients. Angiotensin converting enzyme inhibitors (ACEIs) and angiotensin receptor blockers (ARBs) are widely prescribed as antihypertensive drugs which act on the RAS. Some research indicates that they may be superior to other antihypertensive drugs because the RAS is thought to participate in AD neuropathogenesis via both vascular and amyloid pathways [27]. Kehoe et al. demonstrated that ACE-2 activity is reduced in AD, is an important regulator of the central classical ACE-1/Ang II/AT1R axis of RAS, and that dysregulation of this pathway likely plays a significant role in the pathogenesis of AD [11]. However, recent evidence indicates that Resveratrol treatment decreased Ang II serum level, the aortic expression of prorenin receptor (PRR) and ACE, increased Ang-(1–7) serum level and the expression of ACE2, AT2R, and Mas receptor (MasR) [41]. Besides, Kamel et al. indicated that overactivation of ACE/Ang2/AT1 axis with activated AT1 impairs the survival pathway PI3K/Akt, and triggers amyloid-β-induced apoptosis and neurodegeneration in AD [54]. The Aβ aberrantly regulated caspase-3, GSK-3β, and Akt signalling in the hippocampus. Caspase-3 and GSK-3β inhibitors ameliorated memory impairments and synaptic deficits in Aβ-injected AD model mice. Recent evidence also found that pharmacological activation of Akt rescued memory impairments and aberrant synaptic plasticity in Aβ-injected mice AD [55]. However, our results suggest that resveratrol exerts effects similar to those of the Ang-II receptor antagonist losartan by attenuating Ang-II-induced tau pathologies, improving Akt activation, and attenuating down-regulation of the AT1R–Aβ–caspase 3–GSK-3β signalling pathway (Figure 3). Resveratrol targets the CNS and is able to cross BBB, triggering neuroprotective effects [56]. In addition, Vingtdeux et al. observed that resveratrol is present in the brain following oral administration in patients with neurological disorders [46].

Our results demonstrated that resveratrol treatment for two weeks decreased BP, attenuated ROS production in the NTS, and increased BDNF levels in the hippocampus of Ang-II-induced early AD rats. In addition, losartan inhibition of Tau^T231^ phosphorylation in the hippocampus significantly abolished Ang-II-induced expression of Aβ precursors, active caspase 3, and glycogen synthase kinase 3β (GSK-3β)^Y216^, accompanied with Akt^S473^ phosphorylation. In addition, the effect of losartan is shared by resveratrol. Interestingly, resveratrol reversed in hippocampal-dependent and contextual memory impairments by NOX2 deletion and SOD2 elevation.

In summary, our results show that resveratrol reduces oxidative stress to protect brain from memory impairment and hippocampal damage in early stage AD rat model, however, resveratrol effect is not as strong as losartan. Therefore, these novel findings indicate that resveratrol is a potential pharmacological option similar to losartan for patients with hypertension at risk of AD during old age. Furthermore, our findings provide insights into identification of molecular targets for memory recovery pathway pertaining to AD prevention in patients with hypertension.

## Supplementary Materials

The following are available online at http://www.mdpi.com/2077-0383/7/10/329/s1, Figure S1: BDNF levels were decreased in rats with Ang-II-induced early Alzheimer’s disease.
