# Supplementary material for: Effect of Resveratrol on Reactive Oxygen Species-Induced Cognitive Impairment in Rats with Angiotensin II-Ind uced Early Alzheimer’s Disease
Source: J Clin Med. 2018 Oct 5;7(10):329. doi: 10.3390/jcm7100329 (PMC6210584; doi:10.3390/jcm7100329)
Supplement: Supplementary file 1 [file jcm-07-00329-s001.pdf]

Supplementary Material

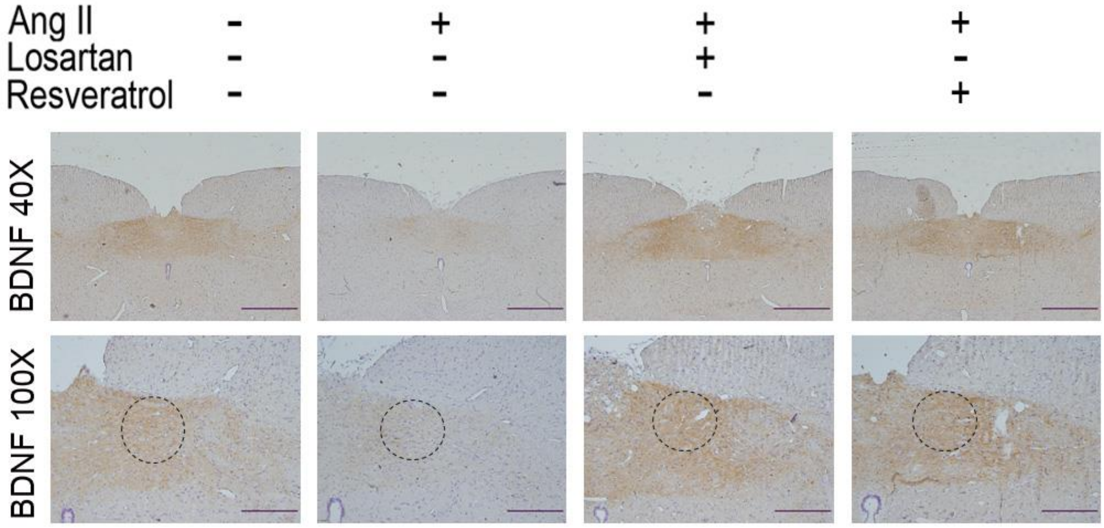

Staining Intensity	Sham	AngII	AngII + Losartan	AngII + Resveratrol
Strong	3	0	3	3
Weak	0	3	0	0
P-value of significant difference between AngII and other groups	0.0182			

Figure S1. BDNF levels were decreased in rats with Ang-II-induced early Alzheimer's disease (AD). *In situ* qualitative analysis of BDNF-immunopositive cells in the nucleus tractus solitarius (NTS) of AD model rats. Scale bar, 200 mm. BDNF-expressing cells after treatment with Ang-II and/or losartan or resveratrol. Note the significant increase in Ang-II-induced BDNF production after the administration of losartan or resveratrol. BDNF: brain-derived neurotrophic factor.
